# Three of a Kind: Control of the Expression of Liver-Expressed Antimicrobial Peptide 2 (LEAP2) by the Endocannabinoidome and the Gut Microbiome

**DOI:** 10.3390/molecules27010001

**Published:** 2021-12-21

**Authors:** Mélissa Shen, Claudia Manca, Francesco Suriano, Nayudu Nallabelli, Florent Pechereau, Bénédicte Allam-Ndoul, Fabio Arturo Iannotti, Nicolas Flamand, Alain Veilleux, Patrice D. Cani, Cristoforo Silvestri, Vincenzo Di Marzo

**Affiliations:** 1 Quebec Heart and Lung Institute Research Centre, Department of Medicine, Faculty of Medicine, Université Laval, Quebec City, QC G1V 0A6, Canada; melissa.shen.1@ulaval.ca (M.S.); claudia.manca.1@ulaval.ca (C.M.); nayudu.nallabelli.1@ulaval.ca (N.N.); Nicolas.Flamand@criucpq.ulaval.ca (N.F.); 2 Unité Mixte Internationale en Recherche Chimique et Biomoléculaire du Microbiome et son Impact sur la Santé Métabolique et la Nutrition, Université Laval, Quebec City, QC G1V 0A6, Canada; 3 Metabolism and Nutrition Research Group, Louvain Drug Research Institute (LDRI), Walloon Excellence in Life Sciences and BIOtechnology (WELBIO), UCLouvain, Université Catholique de Louvain, 1200 Brussels, Belgium; francesco.suriano@uclouvain.be (F.S.); patrice.cani@uclouvain.be (P.D.C.); 4 Centre Nutrition, Santé et Société (NUTRISS), Institut sur la Nutrition et les Aliments Fonctionnels (INAF), École de Nutrition (FSAA), Université Laval, Quebec City, QC G1V 0A6, Canada; florent.pechereau.1@ulaval.ca (F.P.); benedicte.allam-ndoul@criucpq.ulaval.ca (B.A.-N.); Alain.Veilleux@fsaa.ulaval.ca (A.V.); 5 Endocannabinoid Research Group, Institute of Biomolecular Chemistry, Consiglio Nazionale delle Ricerche, 80078 Pozzuoli, Italy; fabio.iannotti@icb.cnr.it; 6 Canada Excellence Research Chair on the Microbiome-Endocannabinoidome Axis in Metabolic Health, Université Laval, Quebec City, QC G1V 0A6, Canada

**Keywords:** endocannabinoid, PPARs, gut microbiome, intestine, ghrelin, LEAP2

## Abstract

The endocannabinoidome (expanded endocannabinoid system, eCBome)-gut microbiome (mBIome) axis plays a fundamental role in the control of energy intake and processing. The liver-expressed antimicrobial peptide 2 (LEAP2) is a recently identified molecule acting as an antagonist of the ghrelin receptor and hence a potential effector of energy metabolism, also at the level of the gastrointestinal system. Here we investigated the role of the eCBome-gut mBIome axis in the control of the expression of LEAP2 in the liver and, particularly, the intestine. We confirm that the small intestine is a strong contributor to the circulating levels of LEAP2 in mice, and show that: (1) intestinal *Leap2* expression is profoundly altered in the liver and small intestine of 13 week-old germ-free (GF) male mice, which also exhibit strong alterations in eCBome signaling; fecal microbiota transfer (FMT) from conventionally raised to GF mice completely restored normal *Leap2* expression after 7 days from this procedure; in 13 week-old female GF mice no significant change was observed; (2) *Leap2* expression in organoids prepared from the mouse duodenum is elevated by the endocannabinoid noladin ether, whereas in human Caco-2/15 epithelial intestinal cells it is elevated by PPARγ activation by rosiglitazone; (3) *Leap2* expression is elevated in the ileum of mice with either high-fat diet—or genetic leptin signaling deficiency—(i.e., *ob*/*ob* and *db*/*db* mice) induced obesity. Based on these results, we propose that LEAP2 originating from the small intestine may represent a player in eCBome- and/or gut mBIome-mediated effects on food intake and energy metabolism.

## 1. Introduction

The liver-expressed antimicrobial peptide 2 (LEAP2) was originally isolated from human hemofiltrates [1]. Its encoding gene is composed of three exons and two introns located at chromosome 5q31 in humans and chromosome 11 in mice. There are two different transcripts of LEAP2 in humans; the one mainly found in the liver, small intestine, kidney, colon, and gastric antrum is the 350bp transcript, whereas, the lung, heart, and trachea express the 550bp transcript [1,2]. The murine *Leap2* RNA encodes for a 76 amino acid protein, although the mature LEAP2 peptide is composed of 40 amino acid residues [1]. The peptide is positively charged and shows similar characteristics to other cationic peptides with antimicrobial activity in vitro. It is composed of an N-terminal (1–14 amino acids) and a C-terminal (15–40 amino acids) domain and two disulfide bonds. LEAP2 shares structural characteristics with antimicrobial peptides such as defensins and LEAP1/hepcidin [1]. Several Gram-positive bacteria, such as *Bacillus megaterium* and *Bacillus subtilis*, are sensitive to treatment with a synthetic LEAP2 peptide, which did not affect the growth of Gram-negative *Escherichia coli* and *Pseudomonas* [1]. The antimicrobial activity of LEAP2 was suggested to be dependent on the amino-terminal domain and not on the induction of membrane destabilization and/or pore formation under physiological conditions [3], indicating that the peptide may bind to an intracellular target to exert this activity.

The strong sequence conservation of LEAP2 in vertebrates might indicate a physiological role unrelated to antimicrobial activity [3,4]. Whilst it was shown that the peptide is not mitogenic for epithelial cells and does not function directly to link the innate and adaptive immune systems [2,5], regulation of the action of ghrelin, a key hormone produced from the stomach and acting in the brain to regulate food intake, reward, and other fundamental central nervous system (CNS) functions, was recently suggested as an additional function of LEAP2. Ge et al. [6] used a mouse model of vertical sleeve gastrectomy (VSG) as a tool to identify secreted proteins and peptides that might act as metabolic regulators. They analyzed various genes that encode for secreted proteins and peptides in the stomach and intestines and found that one set of genes exhibited inverse regulation between the stomach and duodenum. Among these genes, *Leap2* expression increased by 52-fold in the stomach and decreased by 94% in the duodenum following VSG [6]. Subsequently, it was found that LEAP2 acts as an endogenous antagonist of the ghrelin receptor by specifically inhibiting ghrelin binding to its receptor, the GHSR, in a non-competitive manner, thus blocking ghrelin-mediated GH release, food intake, and glucose mobilization [6]. This discovery led to the proposal of LEAP2 as a new potential therapeutic target for uncontrolled ghrelin signaling-related diseases, such as obesity and diabetes, cachexia, anorexia, alcohol abuse, and Prader-Willi Syndrome.

Two other reciprocally interacting players in energy metabolism and its pathological disturbances are: (1) the endocannabinoidome (eCBome), which includes: (a) the endocannabinoids anandamide (AEA), 2-arachidonoylglycerol (2-AG), and noladin ether; (b) the cannabinoid CB1 and CB2 receptors; (c) endocannabinoid-related mediators, such as the *N*-acylethanolamines (NAE), like AEA, and other long-chain fatty acid amides and esters in general, such as the 2-monoacyl-glycerols (2-MAGs), like 2-AG, and the *N*-acyl amino acids, the *N*-acyl-glycines, as well as their receptors (which encompass peroxisome proliferator-activated receptors [PPARs], transient receptor potential [TRP] channels and orphan G-protein-coupled receptors [GPRs]) and anabolic/catabolic enzymes [7]; and (2) the gut microbiome (mBIome), which encompasses thousands of commensal intestinal microorganism species with their armamentarium of genes, proteins and metabolites signaling to the host [8]. Both these systems have been related to ghrelin function, for example through the following mechanisms: (1) ghrelin has been shown to enhance food intake partly via endocannabinoid biosynthesis and CB1 receptor activation, e.g., in the hypothalamus [9,10], while, vice versa, some central effects of CB1 receptors have been suggested to be mediated by activation of the ghrelin receptor, GHSR [11]; additionally, the anorexigenic NAE, *N*-oleoyl-ethanolamine (OEA), which is inactive at cannabinoid receptors and activates instead PPARα, TRPV1 and GPR119, or CB1 activation by endocannabinoids, were suggested to inhibit or stimulate, respectively, ghrelin release from the stomach [12,13,14]; interestingly, in human plasma, similar or opposing alterations in the levels of ghrelin and 2-AG or non-endocannabinoid NAEs, respectively, occur following exposure to palatable food of lean volunteers [15], whereas in obese individuals exposed to chocolate, the plasma levels of both orexigenic and anorexigenic eCBome mediators (i.e., AEA and OEA, respectively) were directly correlated to ghrelin levels [16]; and (2) through some of its specific metabolites, such as the short chain fatty acids (SCFAs), the gut microbiome impacts on ghrelin action at GHSR, whereas concomitant changes in circulating ghrelin levels and specific gut microbiota taxa are also known to occur under different experimental conditions; however, the mechanisms by which the gut microbiota interacts with ghrelin secretion and signaling are still largely unknown [17]. These, and many other previously published data, strongly suggest that both the eCBome and the gut mBIome, through their multiple signaling mechanisms, are likely to regulate energy metabolism also via interactions with ghrelin.

Based on this background, we hypothesized that some of the effects of the eCBome and the gut mBIome on ghrelin action may occur via changes in *Leap2* expression in the liver and, particularly, the intestine, and investigated this hypothesis either in vivo or in vitro. In particular, we first aimed at identifying a direct effect of the gut mBIome by studying *Leap2* expression in germ-free (GF) mice before and after the reinstatement of functional gut microbiota by fecal microbiota transfer (FMT) from conventionally raised (CR) mice. Next, we investigated if the effect of the gut mBIome on *Leap2* expression in the gut was due to its previous effect on the gut eCBome, by looking at how several eCBome mediators, or eCBome receptor activating and inactivating pharmacological tools, affected *Leap2* expression in differentiated intestinal epithelial CaCo-2/15 cells or organoids prepared from the mouse small intestine, in the presence or absence of a gut microbiota-derived pro-inflammatory signal, i.e., lipopolysaccharide (LPS). Finally, we investigated whether, and how, intestinal *Leap2* expression is altered in mouse models of obesity/type 2 diabetes and how such alterations relate to those of the eCBome in the same models, where profound gut microbiota perturbations, sometimes referred to as dysbiosis, also exist.

## 2. Results

### 2.1. Germ-Free Mice Exhibit Altered Levels of Leap2 in the Small Intestine

**Figure 1:**
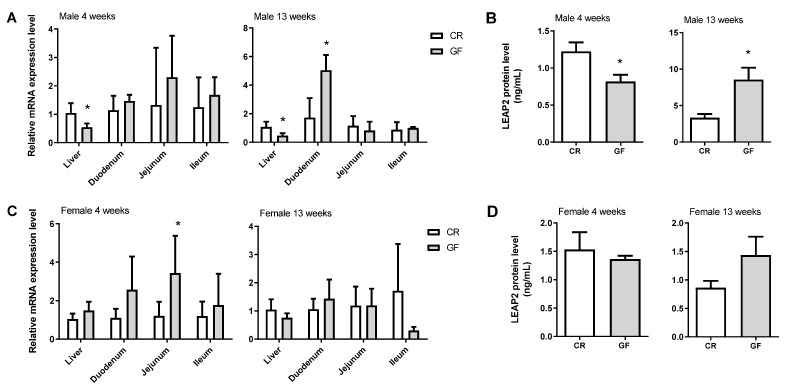
*Leap2* expression in germ-free mice. Gene expression of *Leap2* was measured in the liver and small intestinal regions of conventionally raised (CR) and germ-free (GF) male mice at 4 and 13 (**A**) weeks of age by qPCR and r plotted relative to CR mice for each age and each tissue. LEAP2 protein levels (ng/mL) were measured in the plasma of CR and GF male mice at 4 and 13 (**B**) weeks of age by ELISA and represented as means ± S.D. n = 5–6. * *p* ≤ 0.05. Gene expression of *Leap2* was measured in the liver and small intestinal regions of conventionally raised (CR) and germ-free (GF) female mice at 4 and 13 (**C**) weeks of age by qPCR and plotted relative to CR mice for each age and each tissue. LEAP2 protein levels (ng/mL) were measured in the plasma of CR and GF female mice at 4 and 13 (**D**) weeks of age by ELISA and represented as means ± S.D. n = 5–6. * *p* ≤ 0.05.

We investigated the impact of the gut microbiota on *Leap2* expression in the liver and intestinal sections of both male and female GF mice at 4 weeks and 13 weeks of age as compared to conventionally raised CR mice of the same sex and age (Figure 1). In 4 week-old male mice, we observed a significant decrease in *Leap2* mRNA expression in the liver, and a non-significant increase in the jejunum and ileum, in GF vs. CR mice, whereas there were no trends in the duodenum (Figure 1A). In 13 week-old males, we observed again a significant decrease in *Leap2* mRNA levels in the liver and a significant increase in expression in the duodenum in GF vs. CR mice, whereas, in the jejunum and ileum, there were no changes (Figure 1A). Levels of circulating LEAP2 protein showed a decrease at 4 weeks (Figure 1B), whereas there was a significant increase in 13 week-old male mice (Figure 1B). This suggests that the liver and duodenum, respectively, may contribute the most to LEAP2 circulating plasma levels in 4 and 13-week old male GF mice.

Concerning female mice, *Leap2* mRNA expression showed a tendency to increase at 4 weeks old in GF vs. CR mice in the liver and duodenum, and the same pattern was observed in the jejunum, where there was a significant increase (Figure 1C). In 13 week-old females, there was a non-significant decrease in *Leap2* mRNA expression in the liver and ileum of GF vs. CR mice, whereas there were no observable changes in the other analyzed tissues (Figure 1C). Protein levels of LEAP2 in the blood did not change in either 4 or 13-week old female GF mice (Figure 1D).

**Figure 2:**
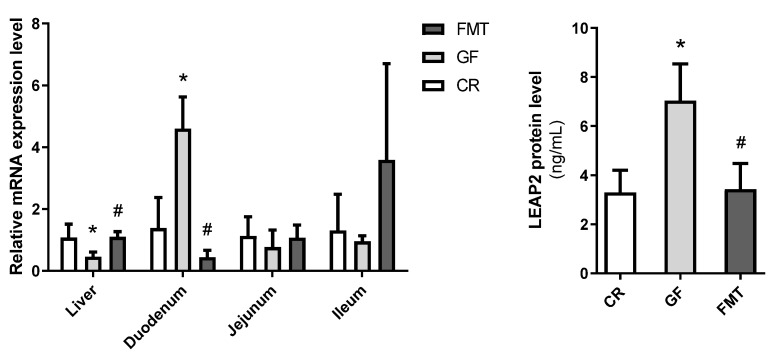
*Leap2* expression in germ-free male mice after a fecal microbiota transfer. Relative *Leap2* gene expression was measured by qPCR (**left**) in the liver and small intestinal regions and LEAP2 protein levels ((**right**); ng/mL) were measured in the plasma by ELISA of 13 week-old conventionally raised (CR) (n = 10), GF (n = 5) and in GF mice after fecal microbiota transfer (FMT) (n = 6). Relative mRNA expression is plotted relative to CR mice for each tissue. Plots represent means ± S.D. Post-hoc analysis was carried out between GF and CR mice to determine the impact of the lack of microbiota or GF and FMT mice to determine the impact of the introduction of microbiota. * GF vs. CR; ^#^ FMT vs. GF. * *p* ≤ 0.05, ^#^ *p* ≤ 0.05.

We next performed a fecal microbiota transfer (FMT) from CR male mice of 13 weeks of age into GF male mice to evaluate whether the reintroduction of the gut microbiota is able to reverse the *Leap2* expression changes observed in GF male mice. For this, we privileged male 12 week-old GF mice, since we saw the most interesting results and changes in older male mice compared to female mice. This procedure was previously reported by us in these same mice to successfully reinstate gut microbiota in GF mice [18]. We first checked if we could repeat the results of the previous experiment, for instance, the decrease in the liver and increase in the duodenum of *Leap2* mRNA expression in GF vs. CR mice, and this was indeed the case. More importantly, in both the liver and duodenum, we found that the FMT was able to revert the changes in *Leap2* expression found in male GF mice, with an increase in the liver and a decrease in the duodenum following FMT (Figure 2A). Circulating protein levels of LEAP2 that were upregulated in 13 week-old male GF mice were likewise reduced to baseline CR mouse levels by FMT (Figure 2B).

These data indicate that the GF status results in sex-dependent significant changes in *Leap2* mRNA expression in the liver and the duodenum, with the latter tissue producing the predominant effect on circulating LEAP2 protein levels in adult male mice, and that such changes are directly due to the lack of the gut microbiota. Since the small intestine of GF mice also exhibits strong FMT-reversible alterations in the levels of eCBome mediators (namely NAEs such as OEA and LEA, which are increased) and receptor mRNAs (namely *Cnr1* and *Ppara*, which are increased, and *Gpr55* and *Gpr18*, which are decreased) [18], we next tested the effect of drugs activating CB1, PPARα, GPR55 and GPR18 receptors on *Leap2* mRNA expression in two different in vitro models of intestinal tissue.

### 2.2. Leap2 mRNA Expression in Organoids from the Mouse Duodenum Is Increased by Noladin Ether

**Figure 3:**
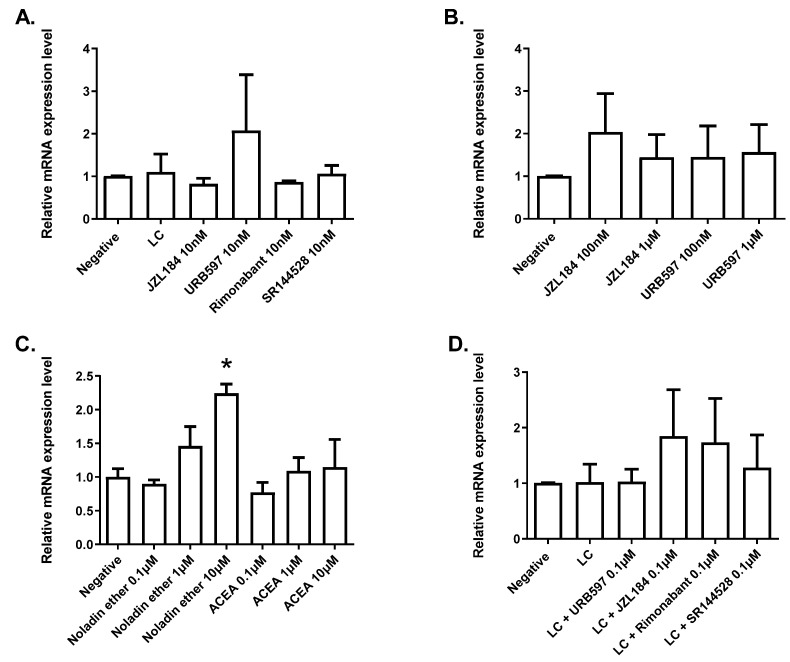
Expression in the duodenum-derived intestinal organoids. Relative *Leap2* gene expression was measured by qPCR in organoids treated with the indicated compounds at the indicated concentrations to test responsiveness to cannabinoid receptor activity through pharmacological manipulation ((**A**–**C**), Rimonabant; CB1 antagonist, SR144528; CB2 antagonist, URB597; FAAH inhibitor, JZL184; MGLL inhibitor) and in response to an inflammatory lipopolysaccharide/cytokine cocktail ((**D**), LC; lipopolysaccharide [LPS, 10 µg/mL], tumor necrosis factor-alpha [TNFα, 100 ng/mL), interleukin 1 beta [IL-1β; 100 ng/mL] and interferon-gamma [IFNγ, 100 ng/mL]) for 24 h and then co-treated with the indicated compound for a further 24 h. Experiments were performed in duplicate or in triplicate with organoids from at least 3 independent organoid cultures (n = 3–5). Each culture was isolated from the duodenum of different mice. Values are represented as mean ± S.E.M and were compared relative to control (Negative; without DMSO). * *p* ≤ 0.005 vs. DMSO.

We have previously reported that several components of the eCBome are modified within the intestinal sections of GF mice, especially within the duodenum [18]. As we observed changes in the expression of *Leap2* in the duodenum of GF mice, we investigated *Leap2* mRNA expression in small intestine organoids isolated from the mouse duodenum and treated with different concentrations of eCBome mediator inactivating enzyme inhibitors, in the absence or presence of a pro-inflammatory cocktail (LPS, TNFα, IL-1β and IFNγ [LC]). In the absence of LC, organoids treated with: (1) MAGL and FAAH inhibitors, i.e., URB597 and JZL184, respectively, which can indirectly activate the targets of NAEs and 2-MAGs; or (2) CB_1_ and CB_2_ antagonists/inverse agonists (rimonabant and SR144528, respectively) at 10 nM, did not exhibit significant alterations in *Leap2* expression (Figure 3A). Higher concentrations of the enzyme inhibitors (100 nM and 1 μM) similarly failed to modify *Leap2* expression (Figure 3B). These data may suggest that MAGL and FAAH do not play a major role in inactivating NAEs and 2-MAGs in small intestine epithelial cells, and/or that there is no endogenous tone by AEA and 2-AG at CB1/CB2 receptors regulating *Leap2* expression within duodenum-derived organoids. In order to confirm this hypothesis, we next tested the effects of the hydrolysis-resistant AEA and 2-AG analogs, ACEA, and noladin ether, respectively, to target CB_1_/CB_2_ receptors directly. Importantly, ACEA and noladin ether, at higher concentrations, are also known to activate TRPV1 and PPARα, respectively [19,20,21]. Treatment with ACEA at different concentrations, 0.1, 1.0, and 10 μM did not change *Leap2* expression (Figure 3C). However, while the two lowest concentrations of noladin ether (0.1 and 1 μM) also did not change *Leap2* expression, the highest concentration (10 μM) resulted in a statistically significant upregulation of more than 2-fold (Figure 3C).

**Figure 4:**
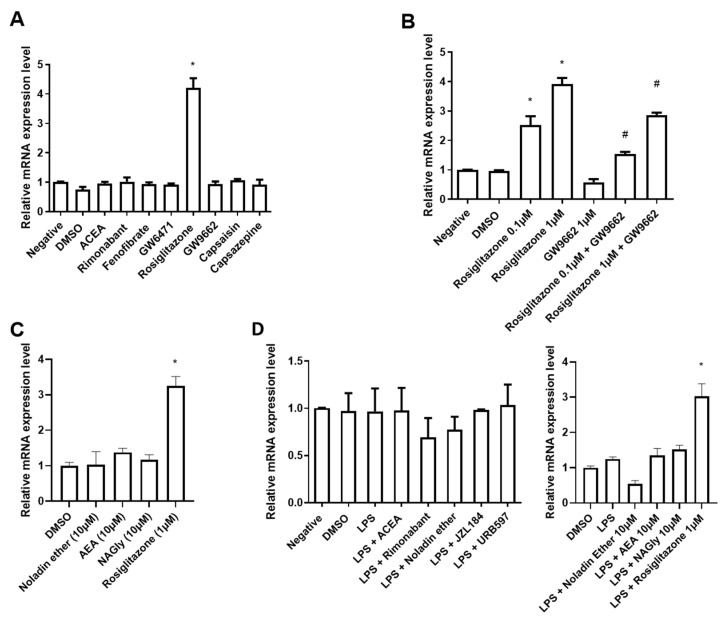
*LEAP2* expression in Caco-2/15 cells. Differentiated cells were treated with the indicated compounds at 1 µM (unless otherwise indicated) for 24 h. Effect of modulation of receptor activity through pharmacological manipulation. ((**A**), ACEA; CB1 and TRPV1 agonist, Rimonabant; CB1 antagonist, Fenofibrate; PPARα agonist, GW6471; PARA antagonist, Rosiglitazone; PPARγ agonist and GW9662; PPARγ antagonist, Capsaicin; TRPV1 agonist and Capsazepine; TRPV1 antagonist). Specificity of *LEAP2* gene expression responsiveness to PPARγ activation (**B**). Effects of noladin ether *N*-arachidonoylethanolamine (AEA) and *N*-arachidonoyl-glycine (NAGly) (**C**). Effects of inflammatory stimulation by lipopolysaccharide (LPS at 10 µg/mL) without and with eCBome pharmacological manipulation ((**D**), URB597; FAAH inhibitor, JZL184; MAGL inhibitor). Values are represented as mean ± S.E.M and were compared relative to DMSO. n = 3. * *p* ≤ 0.05 vs. DMSO; ^#^ *p* ≤ 0.05 vs. relevant Rosiglitazone control.

The LC inflammatory cocktail did not affect *Leap2* expression, nor did co-incubation of LC with URB597, JZL184, SR144528 or rimonabant, all at a 0.1 µM concentration (Figure 4D).

### 2.3. LEAP2 mRNA Expression in Human Intestinal Epithelial Caco-2/15 Cells Is Increased following PPARγ Activation

Duodenal organoids contain all cellular elements of the duodenum, and the epithelial layer in general (except for myenteric neurons). Additionally, the geometry of the organoids is inverse to that of the normal intestinal mucosa, since their basolateral side is outside and directly exposed to treatment, and the apical side is inside and less exposed. Therefore, we wanted to investigate the potential for the eCBome to modulate *LEAP2* expression more selectively in epithelial intestinal cells. To do so, we used the human Caco-2/15 intestinal cell line, an easy model to culture that could give us an insight of how LEAP2 expression might behave in humans, which we first fully differentiated into enterocytes to be treated on their apical side. Differentiated Caco-2/15 cells in monolayers were treated with a 1 μM concentration of agonists and antagonists of eCBome receptors, i.e., ACEA (CB1 and TRPV1 agonist), noladin ether (CB1, GPR55, and PPARα agonist), rimonabant (CB1 antagonist/inverse agonist), fenofibrate (PPARα agonist), GW6471 (PPARα antagonist), capsaicin (TRPV1 agonist), capsazepine (TRPV1 antagonist), rosiglitazone (PPARγ agonist) and GW9662 (PPARγ antagonist), as well as with inhibitors of catabolic enzymes, i.e., URB597 and JZL184. We observed no changes in *LEAP2* expression after treatment with any of these compounds at 1 µM for 24 h, except for a 4-fold increase in enterocytes treated with rosiglitazone (Figure 4A), indicating a possible role of PPARγ. In view of this latter result, we investigated if the effect of rosiglitazone could be abolished using a PPARγ antagonist (GW9662) at 1 µM concentration. We tested two doses (0.1 µM and 1 µM) of rosiglitazone, which dose-dependently increased *LEAP2* expression (Figure 4B), supporting our initial observation (Figure 4A). The PPARγ antagonist alone produced no effect on LEAP2 expression; however, co-treatment with rosiglitazone and GW9662 significantly inhibited the rosiglitazone-mediated increase in LEAP2 expression (Figure 4B). These results indicate that PPARγ is involved in the up-regulation of LEAP2. Since AEA, unlike ACEA, has been suggested to activate PPARγ, at concentrations higher than those required to activate cannabinoid receptors, we tested this compound and found that up to a 10 μM concentration, it did not affect LEAP2 expression, though a trend towards increase was observed (Figure 4C). Finally, *N*-arachidonoyl-glycine (NAGly), which has been proposed as both a PPARα and GPR18 agonist (see above), was also tested at 10 μM and similarly found not to stimulate LEAP2 expression (Figure 4C).

Similar to what was done with organoids, we also tested the impact of inflammation on *LEAP2* expression in enterocytes, and the effect thereupon of pharmacological manipulation of eCBome receptors and enzymes. In this case, the inflammatory state was induced by incubation with only LPS, concomitantly with agonists and antagonists/inverse agonists of eCBome receptors (CB1, PPARγ, PPARα, GPR55, GPR18, TRPV1) and inhibitors (URB597 and JZL184) of the catabolic enzymes (FAAH and MAGL) for 24 h. LPS did not alter LEAP2 expression, either alone or in the presence of any of the compounds, except again for rosiglitazone (Figure 4D). In particular, also, in this case, AEA and NAGly only tended to increase expression in a non-statistically significant manner.

### 2.4. Effect of Noladin Ether on PPARα and PPARγ in a Luciferase Functional Assay

**Figure 5:**
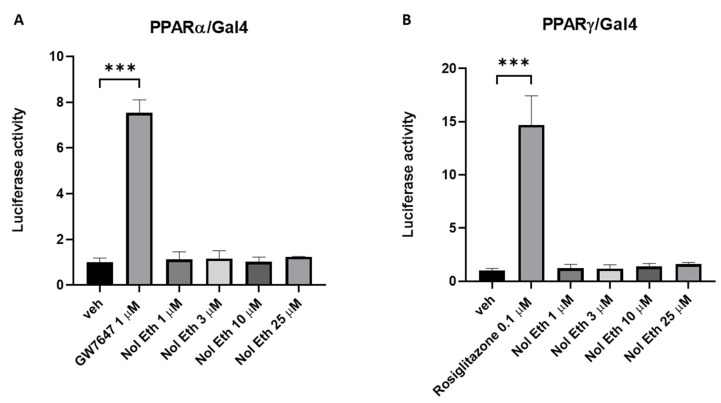
Effect of noladin ether (Nol Eth) in PPARα- or PPARγ reporter cell lines. Luciferase assay was performed in HEK293 cells transiently expressing the ligand-binding domain (LBD) of human PPARα (**A**) and/or PPARγ (**B**) fused to the yeast GAL4 DNA binding domain (DBD). Bar graphs showing the ratio between the firefly and *Renilla* luciferase in response to increasing concentrations of noladin ether. GW7647 (1 µM) and rosiglitazone (0.1 µM) were used as positive controls for PPARα (**A**) and PPARγ (**B**), respectively. The vehicle group value was set to 1. Each point is the mean ± SEM of four separate determinations performed in duplicate. *** *p* ≤ 0.0005 versus the vehicle (dimethyl sulfoxide, DMSO) group.

Due to the stimulatory effect of noladin ether on *Leap2* expression in intestinal organoids and its lack of effect in Caco-2/15 cells, where PPARγ, but not PPARα, activation instead produces elevation of *LEAP2* mRNA levels, and in view of the previous reports of noladin ether as a PPARα agonist, we next wondered if high noladin ether concentrations may be acting through PPARs to affect *LEAP2* mRNA expression in cells other than epithelial enterocytes. In fact, the possibility of PPARα being involved in the noladin ether effect in organoids still exists, since we did not test here PPARα agonists in organoids. To test if high concentrations of noladin ether can activate PPAR-mediated transcription, we performed luciferase assays with this compound at concentrations from 1 to 25 μM. As expected, PPARα/HEK293 and PPARγ/HEK293 cells showed a massive luciferase induction upon exposure to the selective agonists GW7647 and rosiglitazone, respectively. However, no changes were found in either case with noladin ether (Figure 5A,B). Therefore, the mechanism by which noladin ether upregulates *Leap2* expression in duodenal organoids remains to be determined.

### 2.5. Diet-Induced and Genetically Obese Mice Express Altered Intestinal Levels of Leap2 in Relation with Altered Endocannabinoidome Signaling

**Figure 6:**
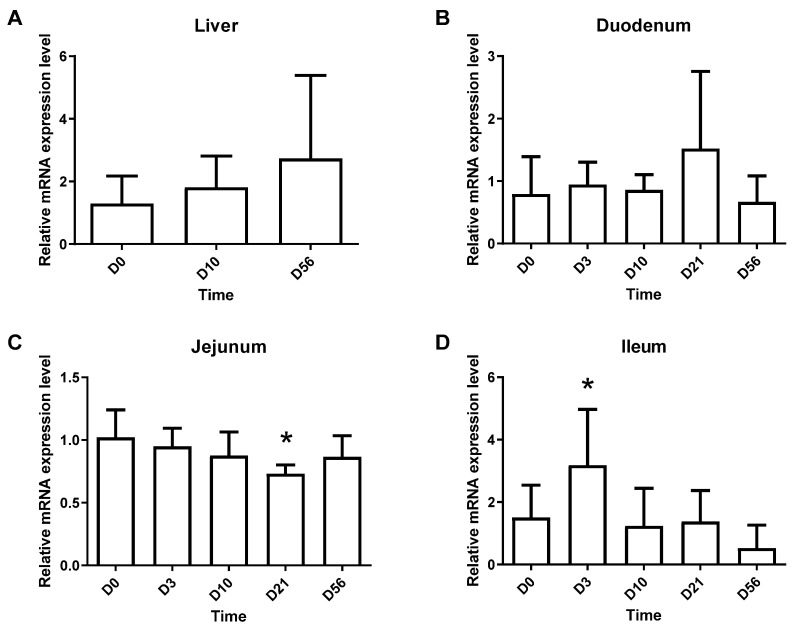
*Leap2* mRNA expression in the liver and small intestine following the initiation of an HFHS diet. Gene expression of *Leap2* in the liver (**A**), duodenum (**B**), jejunum (**C**), and ileum (**D**) at different HFHS diet time points as measured by qPCR. Relative mRNA expression relative to mice at day 0 is represented as mean ± S.D.). n = 10–12. * *p* ≤ 0.05.

It was previously shown that ghrelin levels are altered in a mouse model of diet-induced obesity (DIO) [22]. Here we wanted to explore how DIO affects *Leap2* expression in mice fed with high-fat high sucrose (HFHS) diet at different time points following the beginning of the diet (0 days, 10 days, 21 days, and 56 days). We have previously shown [23] that these same mice progressively gained weight and become glucose intolerant over the duration of the protocol while developing time-dependent alterations in their circulating and intestinal eCBome mediators, enzymes, and receptors [23]. We report a gradual increase in *Leap2* expression in the liver, from day 0 to day 56 that did not reach statistical significance (Figure 6A). Then, we looked at the duodenum and saw no changes in *Leap2* expression, although we observed a non-significant increase on day 21 (Figure 6B). In the jejunum, instead, we observed a significant decrease in *Leap2* expression on day 21, and no changes on days 3, 10, and 56 compared to the baseline (Figure 6C). Finally, in the ileum, we observed a significant increase in *Leap2* expression at day 3, when the mice were already glucose intolerant [23], and a gradual decrease from day 10 to day 56 (Figure 6D), when the mice gradually became obese [23].

**Figure 7:**
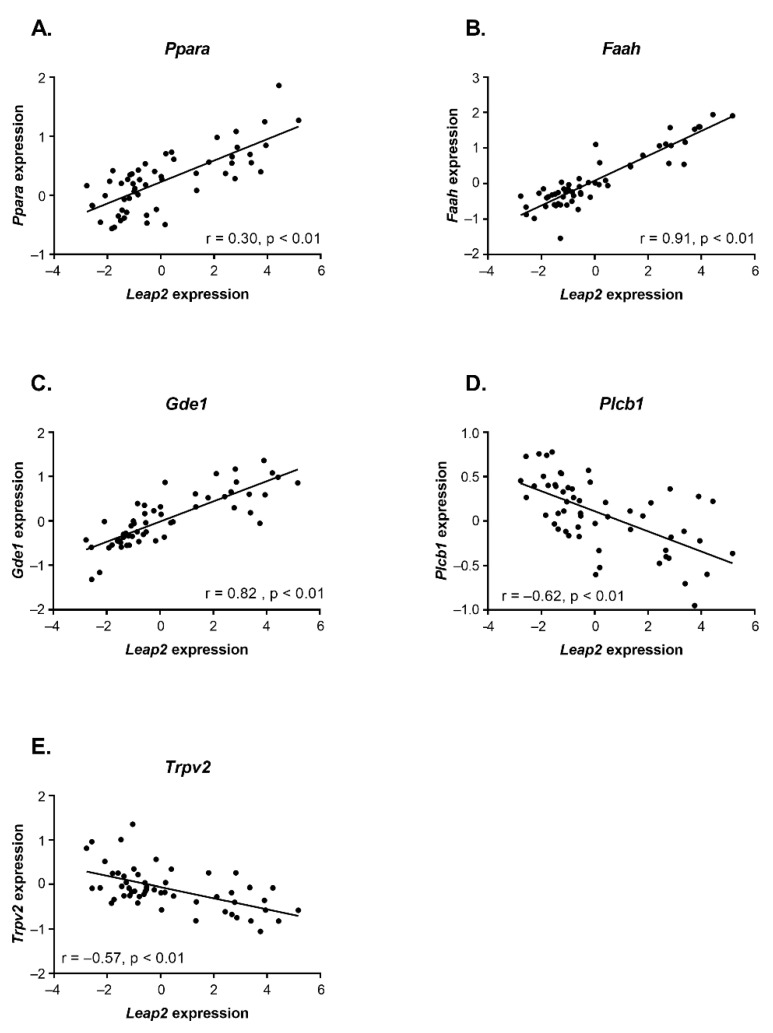
Correlation between the mRNA expression levels of *Leap2* and those of (**A**) *Ppara*, (**B**) *Faah*, (**C**) *Gde1*, (**D**) *Plcb1* and (**E**) *Trpv2* in the ileum of HFHS-fed mice. The figure shows scatterplots with regression lines. Pearson correlation coefficients (r) and *p*-values are shown in graphs (n = 54–56). Data were obtained using the 2-ΔΔCt method relative to the *Tbp* (TATA-binding protein) housekeeping gene (RT-qPCR).

We have reported previously [23] that in the ileum of the same HFHS mice used in the present study the levels of some eCBome mediators, i.e., OEA, 2-oleoyl-glycerol (2-OG), and 2-linoleoyl-glycerol (2-LG), which may act on PPARα, GPR119 and TRPV1 receptors [7], change in a very similar way to what found here for *Leap2* mRNA levels, i.e., they peak at day 3 and then significantly decrease, whereas the levels of the eCB and weak PPARγ agonist, AEA, instead start increasing after 3 days of the HFHS diet. Likewise, the mRNA levels of eCBome genes such as *Faah*, *Abhd6*, and *Ppara* peaked at day 3 before decreasing, whereas *Cnr2*, *Pparg*, and *Plcb1* mRNA levels showed a negative peak at day 3 and then increased with time [23]. Therefore, we wanted to see if the changes we saw in *Leap2* expression in the ileum were correlated with changes in eCBome genes. Interestingly, *Leap2* expression was significantly and positively correlated with the ileal mRNA levels of *Ppara*, *Faah*, and *Gde1*, but not *Abhd6*, and negatively with those of *Plcb1* and *Trpv2*, but not *Cnr2* and *Pparg* (Figure 7). These correlations might suggest the implication of the eCBome, and in particular, of NAEs—which are biosynthesized through GDE1 (among other enzymes) and (like *N*-acyl-glycines) are degraded by FAAH, and act as agonists at PPARα (as in the case of OEA and PEA, similar to *N*-acyl-glycines) and PPARγ (as in the case of AEA) and/or antagonists at TRPV2 (as in the case of OEA and *N*-linoleoyl-ethanolamine [LEA]) [7,24,25]—in *Leap2* up-regulation during the development of HFHS-induced glucose intolerance and obesity. The negative correlation with *Plcb1* might suggest instead the existence of a negative correlation with 2-MAGs, of whose biosynthesis this enzyme catalyzes the rate-limiting step. However, ileal *Leap2* mRNA levels did not correlate with any eCBome mediator levels measured in the ileum (data not shown).

**Figure 8:**
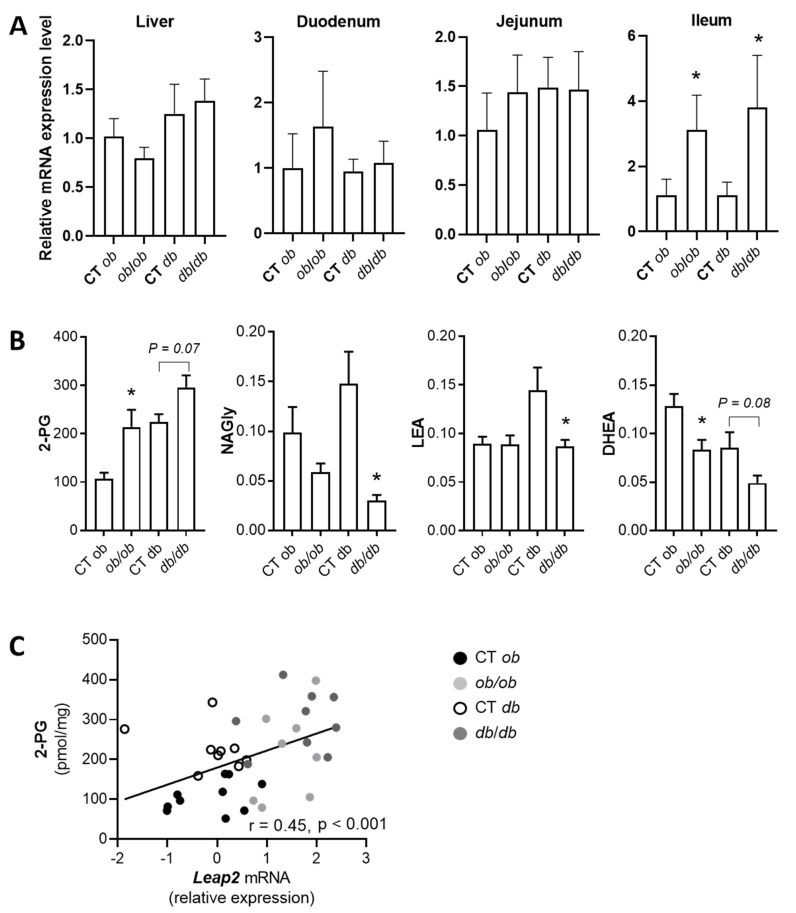
mRNA expression (**A**) in the liver, duodenum, jejunum, and ileum of *ob*/*ob* and *db*/*db* mice as measured by qPCR. Relative mRNA expression is represented as mean ± S.D. Values were compared relative to control (i.e., CT *ob*; CT *db*) mice for each tissue. Levels of select eCBome mediators within the ileum of *ob*/*ob* and *db*/*db* mice and their respective controls ((**B**); pmol/mg wet tissue weight; 2-palmitoylglycerol; 2-PG, *N*-Arachidonoyl-glycine; NAGly; *N*-linoleoylethanolamine; LEA, *N*-docosahexaenoylethanolamine; DHEA), as measured by HPLC-MS/MS. Data are presented as the mean ± S.E.M. of n = 8–10 and were analyzed by one-way ANOVA followed by Tukey’s post hoc test. * *p* ≤ 0.05 vs. the respective control. (**B**). Pearson correlation analysis between the mRNA expression of *Leap2* and the concentration of 2-PG both measured/quantified in the ileum of *ob/ob* and *db/db* mice, and their respective controls (**C**). n = 8–10 mice.

We next analyzed two genetic models of obesity: leptin gene mutant (*ob/ob*) mice and leptin receptor mutant (*db/db*) mice. Although there were trends for a decrease in the liver and for increases in the jejunum and duodenum of *ob/ob* mice, the only significant difference was found in the ileum, where there was a strong increase in *Leap2* expression in both *ob/ob* and in *db/db* mice compared to the corresponding controls (Figure 8A). The results observed in these and DIO mice may suggest that *Leap2* expression in the small intestine, and the ileum, in particular, maybe due to glucose intolerance rather than obesity *per se*, since this dysmetabolic feature was common to both genetically obese mice and HFHS mice when they showed increase *Leap2* mRNA levels.

Finally, also in the case of *ob/ob* and *db/db* mice we looked at possible correlations between the mRNA expression of *Leap2* and eCBome signaling in the ileum. First, we observed that some mediators, such as 2-PG, were also higher in *ob/ob* and *db/db* mice as compared to their respective controls. Other mediators, i.e., NAGly, LEA, and *N*-docosahexaenoylethanolamine (DHEA), instead were significantly, or tended to be, lower in the *ob/ob* and *db/db* mice when compared to the respective controls (Figure 8B). Accordingly, we found that the levels of 2-PG, a PPARα agonist [26], positively correlated with *Leap2* mRNA expression (Figure 8C), which however did not correlate with any of the mRNAs of eCBome genes (data not shown).

In summary, the results obtained in animal models of glucose intolerance and obesity suggest that ileal *Leap2* expression may represent an early adaptive response aimed at counteracting dysmetabolism. This response might be under the indirect positive control of PPARα and its eCBome agonists (as suggested by the positive correlations with *Ppara*, the NAE synthesizing enzyme *Gde1*, and AEA and *N*-acyl-glycine degrading enzyme *Faah*, as well as 2-PG, which is also a good PPARα agonist). Based on the results of the previous section, however, such control would not occur in enterocytes, where instead PPARγ activation may play a role.

## 3. Discussion

LEAP2 is a recently discovered, a potential endogenous regulator of food intake and energy metabolism through antagonism of ghrelin action [6]. In agreement with these properties of this peptide, originally isolated from the liver as an antimicrobial agent, are the following previous findings: (1) selectively in female mice subjected to a high-fat diet, *Leap2* deletion raises body weight, food intake, lean mass, and hepatic fat, and reduces O_2_ consumption, heat production, and locomotor activity during the first part of the dark period [27]; (2) in both female and male lean mice, *Leap2* deletion renders the animals more sensitive to the hyperphagic actions of ghrelin [27]; LEAP2 administration also reduces blood glucose levels in lean mice [6]; (3) in humans and mice, circulating LEAP2 levels display an inverse pattern compared to ghrelin, by increasing with food intake and obesity, and decreasing upon fasting and weight loss [28,29]; refeeding decreases circulating levels of ghrelin, while LEAP2 goes up to baseline levels; and (4) like other anorectic signals, LEAP2 is also produced by the small intestine [2]. Others have reported that all regions of the mouse small intestine express *Leap2*, with the highest expression having been found in the jejunum (whereas we detected slightly higher levels in the duodenum), with next to no detectable levels expressed in the stomach [6]. Regardless, after bariatric surgery (vertical sleeve gastrectomy), the expression in the jejunum decreased significantly, while expression in the stomach increased over 50-fold. This shows that *Leap2* expression can respond to various experimental paradigms in an intestinal-region-specific manner. However, LEAP2 regulation in this tissue has not yet been investigated, nor have its potential interactions therein with two major and inter-related players in obesity, i.e., the eCBome and the gut mBIome. Here we have reported evidence suggesting that: (1) the presence or absence of the gut microbiota strongly and directly affects *Leap2* mRNA expression in the liver and duodenum, and LEAP2 circulating levels, in 13 week-old male, but not female, mice (gender differences are known to influence gene expression [30] and their targeting by transcription [31] and translation regulators that ultimately lead to different mRNAs or protein products [32]); (2) despite the fact that the gut microbiota also strongly and directly affects small intestinal eCBome signaling, generalized modulation of eCBome receptors does not result in significant changes of *Leap2* expression in a model of human enterocytes, except for the activation by the synthetic (i.e., rosiglitazone) agonist of PPARγ, which caused a strong upregulation; additionally, noladin ether, another endocannabinoid discovered by Raphael Mechoulam and his group in 2001 [33], caused a significant elevation of *Leap2* expression in organoids from the mouse duodenum, through yet to be investigated mechanisms; and (3) *Leap2* expression in the ileum of HFHS- and genetically (leptin signaling deficiency)-induced obesity is increased concomitantly with glucose intolerance rather than obesity, and in a manner variedly and strongly correlated with the ileal levels of either an eCBome mediator (2-PG, positive) or mRNAs of proteins that participate in either NAE or 2-MAG biosynthesis (*Gde1*, positive; *Plcb1*, negative) or actions (*Ppara*, positive; *Trpv2*, negative), or in NAE and *N*-acyl-glycine inactivation (*Faah*, positive).

The gut microbiota is known to affect host physiology, and metabolism in particular, through a plethora of microorganism-derived molecules, of which eCB-like mediators different from those produced by the host are also part [34]. Therefore, it was not surprising to find previously that the gut microbiota is a strong determinant of eCBome signaling in the mouse intestine [18], although this action of intestinal microorganisms could also be mediated by other, non-eCBome-related molecules, such as, for example, short-chain fatty acids, tryptophan metabolites and secondary bile acids [35,36]. Likewise, the tonic inhibitory effect of the gut microbiota on duodenal *Leap2* expression, described here for the first time in adult male mice, might be both eCBome- and non-eCBome-mediated. It is noteworthy that the intestinal mBIome is markedly varied along the length of the gastrointestinal tract, with there being relatively few bacteria in the duodenum, and the number and diversity of bacteria increasing distally [37,38]. This of course does not discount the ability of the duodenal microbiome, by being significantly modified with obesity [38,39], to impact both the host physiology and small intestinal bacterial overgrowth [40]. Indeed, we have reported that fecal microbiota transfer into germ-free mice reconstitutes the small intestinal bacterial communities and significantly modulates the eCBome of the small intestine, including the duodenum [18].

We found that at least one eCBome mediator, noladin ether, can stimulate *Leap2* expression in duodenal organoids, which, together with the previous observation that the gut microbiota tonically reduces duodenal PPARα expression in the small intestine of adult male mice [18], may suggest that this receptor and its endogenous ligands might mediate in part gut microbiota tonic inhibition of duodenal *Leap2* expression. However, we could not confirm the proposed mechanism of action of noladin ether in organoids, as this compound did not activate recombinant PPARγ or PPARα in a functional assay, nor could we show that it stimulates *Leap2* expression in a model of human enterocytes. Additionally: (1) PPARγ (an alternative AEA target), although involved in stimulating *Leap2* expression in vitro, particularly when rosiglitazone was used as an agonist, is not significantly altered by the lack of presence of the gut microbiota in terms of mRNA expression in the duodenum [18]; (2) GPR55 (another proposed target for AEA and noladin ether) and GPR18 (a proposed target for NAGly) are tonically stimulated, rather than inhibited, in the duodenum by the gut microbiota [18], and hence cannot partake in microbiota tonic inhibition of *Leap2* expression; and (3) also the duodenal expression of *Cnr1* or *Cnr2*, the two preferential targets for eCBs, is not significantly altered by the gut microbiota [18]. In sum, present and previous data suggest that the effects of the gut microbiota on *Leap2* expression and eCBome signaling might be two unrelated, or only very partially related, phenomena. Nevertheless, the increase in *Leap2* expression observed in GF mice might still account, at least in part, for their more favorable metabolic and glycemic profile under conditions of DIO [41].

Indeed, we observed here, again for the first time, that increased RNA levels encoding for this peptide are produced in the ileum 3 days following an HFHS diet, concomitantly with the appearance of hyperglycemia, and in both *ob/ob* and *db/db* mice that, apart from being obese, are also glucose intolerant. This finding may suggest that LEAP2 is overproduced by the ileum to counteract glucose intolerance induced by an HFHS diet or hyperphagia. Since the ileum of HFHS and of *ob/ob* and *db/db* mice presents with inter-related alterations in gut eCBome signaling (present results and [23]) and microbiota composition [23,42,43], we hypothesized a role of either system in the modulation of *Leap2* expression. However, also, in this case, the only potential eCBome signaling pathway that changed in a manner similar to *Leap2* expression following HFHS and in *ob/ob* and *db/db* mice was that mediated by PPARα and/or its ligands. Conversely, as mentioned above, the only eCBome signaling pathway that was clearly implicated in *Leap2* expression stimulation in human enterocytes was that mediated by PPARγ, whose expression, however, peaks negatively in HFHS mice when *Leap2* expression peaks positively. This may suggest that also in the case of obesity and hyperglycemia the regulation of *Leap2* expression and eCBome signaling might be two unrelated phenomena, and not controlled in a coordinated manner by gut dysbiosis.

A limitation of this study is that the selected in vitro systems for the small intestine do not necessarily reflect what is going on in vivo in the ileum. The organoids were prepared from the duodenum and not from the ileum, and therefore results obtained in this system may only be relevant to those in GF mice, where the strongest reduction in *Leap2* expression was indeed found in this small intestinal section. Yet, the geometry of organoids is such that their basolateral side, rather than the apical one, is the one that is exposed to treatments, which may have altered the effects of the latter. Differentiated Caco-2 cells, instead, model only one cell type (enterocytes) among the several ones that are found in the small intestinal mucosa. Therefore, the findings in these two in vitro systems may not necessarily be relevant to the actual in vivo regulation of *Leap2* expression by the gut microbiota in the dysbiosis typical of GF and hyperglycemic/obese mice, thus leaving still open the possibility that the eCBome, *per se* or following its modulation by the gut microbiome, might be an important determinant of LEAP2 production. In this sense, it is noteworthy that the mRNA expression of this potentially metabolically beneficial endogenous antagonist of ghrelin action is stimulated in vitro by, and correlates in vivo with, non-CB1-mediated (and hence non-metabolically “noxious”) eCBome signaling, i.e., respectively: (1) activation of PPARγ, which is known to be an intermediate in anti-diabetic and anti-glucose intolerance drugs, and (2) expression of PPARα and the levels of one of its endogenous agonists, i.e., 2-PG, with potential anorectic and anti-dyslipidemic actions.

In summary, we have provided here unprecedented evidence for the existence of gut microbiota tonic in vivo control over intestinal *Leap2* expression, which persists into adulthood, at least in the duodenum, of male mice. We also provided preliminary in vitro data suggesting that stimulation of the levels of the mRNA encoding for this metabolically beneficially peptide may be exerted by non-CB1-mediated eCBome signaling. It remains to be clarified whether the gut microbiota-eCBome axis is involved in the control of intestinal LEAP2 levels, especially during obesity and hyperglycemia, where, as shown previously and confirmed here, all the members of this triangle undergo adaptive regulation.

## 4. Materials and Methods

### 4.1. Animals and Housing

Conventionally Raised (CR) and Germ-free (GF) C57BL/6NTac mice were purchased from Taconic (Taconic Bioscience, NY, USA) and maintained in the animal facility of the Institut Universitaire de Cardiologie et Pneumologie de Québec (IUCPQ, QC, Canada). All animals have grouped 3–4 mice per cage under a 12 h:12 h light-dark cycle with ad libitum access to NIH-31 Open Formula Autoclavable Diet (Zeigler, PA, USA) and water. GF mice were housed in axenic status and fecal samples were weekly tested for microbes and parasites by the facility’s staff to ensure that the GF unit was indeed sterile. Both GF and CR mice were acclimatized for at least one week prior to starting the procedures.

### 4.2. Animal Experiments and Fecal Microbial Transplant (FMT)

Twelve (6 male and 6 female) CR and GF mice at 4 and 13 weeks of age, were intraperitoneally anesthetized with a cocktail of ketamine/xylazine/acepromazine at a dose of 50/10/1.7 mg/kg body weight and euthanized by cervical dislocation, following an intra-cardiac puncture. Whole blood was collected in K3-EDTA tubes. The abdominal cavity was opened and the whole digestive tract was carefully aligned from the stomach up to the colon. Once the stomach was removed, the small (duodenum, jejunum, ileum) and large (cecum and colon) intestine were carefully excised and separated and the intestinal contents were harvested by flushing with 1ml of sterile PBS without Ca/Mg (Thermo Fisher Scientific, MA, USA) and snap-frozen. The liver was also isolated from the abdominal cavity. Sections of the liver as well as small and large intestine were stored either in RNALater (Thermo Fisher Scientific, MA, USA) for RNA stabilization or immediately snap-frozen and stored at −80 °C for further analysis. This common procedure was concluded, for each mouse, within a maximum of 15 min, to ensure the preservation of mRNA and lipid for further analysis.

For fecal microbiota transplant (FMT) experiments only 12 weeks old male mice were utilized. GF mice were randomly divided into two groups at the age of 12 weeks: those gavaged with sterile PBS (SHAM; 5 mice) and those gavaged with fecal material (FMT; 6 mice). Material gavaged for FMT consisted of a cocktail of the intestinal contents and stools of a single and 4 CR donor mice, respectively. Briefly, the intestinal contents of the duodenum, jejunum, ileum, colon, and cecum were collected from one 12-week-old CR donor mouse and mixed with stool pellets from all the CR mice to be used as controls. The mixture was well homogenized, weighed, suspended at 1:10 in sterile PBS, and centrifuged at 805× *g* for 10 min at room temperature. The supernatant was used to gavage the mice (200 µL of homogenate per mouse) [18]. The FMT mice were then housed (3 per cage, like for CR mice) for one week in conventional conditions in cages contaminated with used litter coming from donor mouse cages. SHAM mice were submitted to a similar gavage with saline solution, but then kept in the germ-free facility for a week in the same conditions as GF mice. CR mice were euthanized the day of the gavages, while SHAM and FMT mice were sacrificed one week after the gavage; the tissues were collected from all animals as previously described.

### 4.3. RNA Isolation, Reverse Transcription, and qPCR

RNA was extracted from tissues and cells with the RNeasy Plus Mini Kit (Qiagen, Hilden, Germany) following the manufacturer’s instruction and eluted in 50 μL of UltraPure Distilled Water (Invitrogen, CA, USA). The concentration and purity of RNA were determined by measuring the absorbance of the RNA in a Biodrop at 260 nm and 280 nm, and RNA integrity was assessed by an Agilent 2100 Bioanalyzer, using the Agilent RNA 6000 Nano Kit (Agilent Technologies, CA, USA). 1 µg of total RNA was reverse transcribed using the iScript cDNA synthesis kit (Bio-Rad, CA, USA) in a reaction volume of 20 μL. *Leap2* mRNA expression levels were determined using primer pairs (Mm.PT.58.6158455.g, IDT, IA, USA) on a CFX384 touch qPCR System (BioRad) using PowerUp SYBR Green qPCR master mix (Thermo Fisher Scientific, CA, USA) in duplicate reactions. Hprt1 (Mm.PT.39a.22214828, IDT, IA, USA) was used as a reference gene. Because the Ct of the reference gene was the same in all the 13 weeks old control samples (first and FMT experiments), we merged the two control sets into 13 weeks control group. Gene expression levels in mice and cells (see below) were evaluated by the 2-ΔΔCt method and represented as fold increase with respect to the baseline of the relevant control. Data were analyzed by ANOVA followed by Dunnett’s multiple comparisons.

### 4.4. Organoid Preparation and Treatment

Crypt-derived organoids were mechanically separated from sacrificed black male C57BL/6 mouse duodenum. Murine small intestines were opened longitudinally, scratched, and washed with cold phosphate-buffered saline (PBS) (Gibco Life Technologies, Carlsbad, CA, USA). Organoids were incorporated in a solid matrix (Corning^®^ Matrigel^®^) (Corning, Corning, NY, USA) and were incubated with advanced DMEM media (Dulbecco’s Modified Eagle’s Medium) (Gibco Life Technologies, Carlsbad, CA, USA) supplemented with HEPES buffer (Thermoscientific, Waltham, MA, USA), Glutamine (GlutaMAX) (Gibco Thermoscientific, Waltham, Ma, USA), antibiotic Pen-Strep (Millipore Sigma, Oakville, ON, Canada), Noggin (Millipore Sigma, Oakville, ON, Canada), mRSPO (Peprotech, Cranbury, NJ, USA), B27 supplement (Gibco Life Technologies, Carlsbad, CA, USA), EGF (Cedarlane, Burlington, ON, Canada, and N-acetyl-cystein (Millipore Sigma, Oakville, ON, Canada) and Ly-27. Organoids were split 1 into 3 every week, in 24 wells plates, and media was changed every 2 days. on Mature organoid cultures, 4 days following plating, were exposed 24 h to treatment or vehicle.

### 4.5. Caco-2/15Cell Differentiation and Treatment

Human Caco-2/15 intestinal cells (kindly provided by Dr. Jean-François Beaulieu (Université de Sherbrooke, Sherbrooke, Canada) were cultured in high glucose Dulbecco’s Modified Eagle Medium (DMEM) supplemented with 10% Fetal Bovine Serum (FBS, Neuromics), 10 mM HEPES, 1X GlutaMAX, and 100 U/mL Penicillin/Streptomycin. Cell lines were grown until confluence, split, and plated 2 × 10^5^ cell/well for differentiation for 21 days to induce the enterocyte phenotype. On the day of the experiment (after 21 days of differentiation), cells were treated for 24 or 48 h with media containing compounds at the indicated concentrations. DMSO controls were at 0.1%. For the LPS induction, 24 h prior to the experiment, cells were treated with 10ug/mL of LPS (Sigma) before being incubated for 24 h with the indicated compounds at the indicated concentrations. After incubation, media was removed, cells were washed with 1X PBS and frozen for RNA extraction as above.

### 4.6. PPAR-Luciferase Assays

Human embryonic kidney 293 cells (ATCC CRL-1573) were propagated in a growth medium (GM) composed of Dulbecco’s modified Eagle’s medium (DMEM cat. n. 41966029; Thermo Fisher, Monza, Italy) supplemented with 10% fetal bovine serum (cat. n. 16000044; Thermo Fisher, Monza, Italy) and 1% Pen/Strep (cat. n. 15140122 Thermo Fisher, Monza, Italy) under standard conditions. After plating (in 24 well plate density; 5 × 104 cells/well), the cells were transfected on the next day with the following plasmids: (a) pM1-hPPARα-Gal4 or pM1-hPPARγ-Gal4; (b) TK-MH100 × 4-Luc containing the UAS enhancer elements and; (c) Renilla luciferase (pRL, Cat. E2231; Promega, Milan, Italy) using lipofectamine 2000 (cat. n. 11668027; Life Technologies; Milan, Italy). The next day, the growth media was replaced with fresh media containing vehicle (Dimethyl sulfoxide, DMSO ≤ 0.03%) or compounds of interest (noladin ether Cat. No. 1411, GW7647 Cat. No. 1677/10 and Rosiglitazone Cat. No. 5325/10, purchased from TOCRIS, Abingdon, UK). On day 3, the cells were harvested and processed for analysis of luciferase activity using a GloMax Luminometer instrument (Promega, Milan, Italy) and the Dual-Luciferase Reporter Assay kit (cat. n. E1910 Promega, Milan, Italy) following published procedures [26].

### 4.7. Lipid Extraction and HPLC-MS/MS for the Analysis of eCBome Mediators

Lipids were extracted from ileum samples as previously described [18,23,26]. Briefly, about 10 mg of ileum was sampled and homogenized in 1 mL of a 1:1 Tris-HCl 50 mM pH 7: methanol solution containing 0.1 M acetic acid and 5 ng of deuterated standards. One ml of chloroform was then added to each sample, which was then vortexed for 30 s and centrifuged at 3000× *g* for 5 min. The organic phase was collected and another 1 mL of chloroform was added to the inorganic one. This was repeated twice to ensure the maximum collection of the organic phase. The organic phases were pooled and evaporated under a stream of nitrogen and then suspended in 50 µL of mobile phase containing 50% of solvent A (water + 1 mM ammonium acetate +0.05% acetic acid) and 50% of solvent B (acetonitrile/water 95/5 + 1 mM ammonium acetate +0.05% acetic acid). Forty μL of each sample were finally injected onto an HPLC column (Kinetex C8, 150 × 2.1 mm, 2.6 μm, Phenomenex) and eluted at a flow rate of 400 μL/min using a discontinuous gradient of solvent A and solvent B [18,23,26]. Quantification of eCBome-related mediator, was carried out by HPLC interfaced with the electrospray source of a Shimadzu 8050 triple quadrupole mass spectrometer and using multiple reaction monitoring in positive ion mode for the compounds and their deuterated homologs [18,23,26].

### 4.8. Experiments in Mice Undergoing a High Fat High Sucrose Diet

As previously described [20], sixty 6-week-old C57BL/6J male mice were fed ad libitum with a low-fat, low-sucrose purified diet (10% fat and 7% sucrose [LFLS]; Research Diet, NJ, USA) for a 10-day acclimatization period in the animal facility of the Institute of Nutrition and Functional Foods. Mice were then randomly assigned to 6 groups (n = 12) fed a high-fat, high-sucrose purified diet (45% fat and 17% sucrose [HFHS]; Research Diet, NJ, USA) for up to 56 days. These dye-free diets harbor comparable fiber contents, and while the HFHS diet had, by design, a higher fatty acid content, the omega-3/omega-6 ratios were comparable. Six-hour-fasted mice were sacrificed by cardiac puncture to retrieve plasma (1780× *g*, 10 min) at either 0 (baseline), 3, 10, 21, or 56 days following HFHS diet initiation. Duodenum was collected 2 cm of the pylorus, while jejunum and ileum were collected 10 cm and 2 cm, respectively, from the ileocecal junction. All samples were stored at −80 °C until batch analysis.

### 4.9. Experiments in ob/ob and db/db Mice

As previously described [36], male homozygous *ob*/*ob* mice (B6.V-Lepob/ob/JRj) were used as a leptin-deficient obese model, and their lean littermates served as controls (CT ob); (n = 9–10 per group). Male homozygous *db*/*db* mice (BKS-Lepr/db/db/JOrlRj) functionally deficient for the long-form leptin receptor were used as a hyperleptinemic obese type 2 diabetic model, and their lean littermates served as controls (CT db); (n = 9–10 per group). Mice were purchased at the same time and from the same supplier (Janvier Laboratories, Le Genest-Saint-Isle, France) at the age of 6 weeks. Mice were housed in a specific pathogen and opportunistic free (SOPF) controlled environment (room temperature of 22 ± 2 °C, humidity 55 ± 10%, 12 h daylight cycle, lights off at 6 p.m.) in groups of two mice per cage, with free access to sterile food and sterile water. Upon delivery, mice underwent an acclimation period of one week, during which they were fed a standard diet containing 10% calories from fat (D12450Ji; Research Diet; New Brunswick, NJ, USA) and were then kept ad libitum on the same diet for 7 weeks. Milli-Q water filtered by a Millipak^®^ Express 40 with a 0.22 µm membrane filter (Merck Millipore, Burlington, MA, USA) was autoclaved and provided ad libitum. All mouse experiments were approved by and performed in accordance with the guideline of the local ethics committee (Ethics committee of the Université Catholique de Louvain for Animal Experiments specifically approved this study that received the agreement number 2017/UCL/MD/005). Housing conditions were specified by the Belgian Law of 29 May 2013, regarding the protection of laboratory animals (agreement number LA1230314).

## Sample Availability

All compounds used are commercially available.
